# *LINC00857* Interacting with YBX1 to Regulate Apoptosis and Autophagy via MET and Phosphor-AMPKa Signaling

**DOI:** 10.1016/j.omtn.2020.10.025

**Published:** 2020-10-22

**Authors:** Wenmei Su, Lihui Wang, Huijie Zhao, Shengmin Hu, Yi Zhou, Chunfang Guo, Bin Wu, Lixia Li, Zhixiong Yang, David G. Beer, Guoan Chen

**Affiliations:** 1 Department of Pulmonary Oncology, Affiliated Hospital of Guangdong Medical University, Zhanjiang, China; 2 Key Laboratory of Longevity and Aging-Related Diseases of Chinese Ministry of Education, Center for Translational Medicine & School of Preclinical Medicine, Guangxi Medical University, Nanning, China; 3 School of Medicine, Southern University of Science and Technology, Shenzhen 518055, China; 4 Department of Surgery, University of Michigan, Ann Arbor, MI, USA

**Keywords:** *LINC00857*, YBX1, lung cancer, autophagy, apoptosis

## Abstract

Long noncoding RNA (lncRNA) *LINC00857* has been reported to be upregulated in lung cancer and related to poor patient survival. It can regulate cell proliferation and tumor growth in lung cancer as well as several other cancers. However, the underlying molecular mechanisms that are regulated by *LINC00857* are unclear. In this study, we found that *LINC00857* silencing can impair cell proliferation in 14 different genomic alterations of lung cancer cell lines. These alterations are *EGFR*, *KRAS*, *TP53*, *MET*, and *LKB1* mutations. The cell apoptosis and autophagy were induced upon *LINC00857* silencing in lung cancer cells. Mechanistically, *LINC00857* can bind to the Y-box binding protein 1 (YBX1) protein, prevent it from proteasomal degradation, and increase its nuclear translocation. *LINC00857* regulated MET expression via YBX1 at a transcriptional level. Induced cell autophagy by *LINC00857* knockdown was mainly through increased phosphor-AMP-activated protein kinase (p-AMPK)a. Collectively, *LINC00857-*YBX1-MET/p-AMPKa signaling is critical to regulate cell proliferation, apoptosis, and autophagy, which may provide a potential clinically therapeutic target in lung cancer.

## Graphical Abstract

**Figure:**
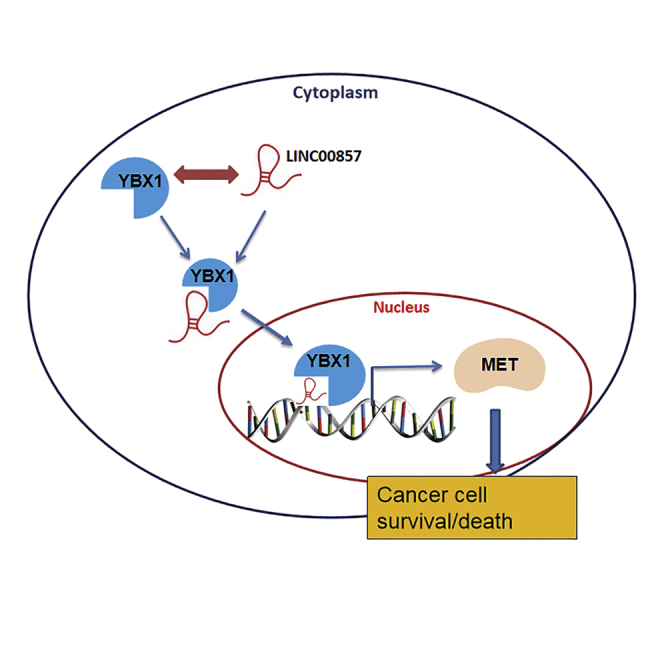

## Teaser

Su and colleagues demonstrated that *LINC00857* silencing can decrease cell proliferation and induce apoptosis and autophagy in lung cancer cells. *LINC00857* is a potential oncogene that acts through binding and stabilizing YBX1, thereby regulating cell survival/death via YBX1-MET and p-AMPKa signaling.

## Introduction

Based on the Global Cancer Observatory (GLOBOCAN) statistics, lung cancer is the most frequent cancer (2.094 million in 2018) and the leading cause of cancer-related death worldwide (1.761 million deaths in 2018).^1^, ^2^, ^3^ Pathologically, lung cancer is classified into two major subtypes: small cell lung carcinoma (SCLC) and non-small cell lung cancer (NSCLC). NSCLCs comprise 85% of all lung cancer cases and are subclassified into adenocarcinoma (AD), squamous cell carcinoma (SCC), and large cell carcinoma.^4^^,^^5^ Although new approaches have emerged during past decades in the treatment of lung cancer patients, including reagents that target epidermal growth factor receptor (EGFR) mutation, alkaline phosphatase (ALK) fusion, and programmed death ligand 1 (PD-L1) immunotherapy,^6^, ^7^, ^8^, ^9^ the 5-year survival rate of NSCLC remains less than 18%.^2^^,^^3^ The poor prognosis and high recurrence rate of lung cancer may be partially due to the histological and molecular heterogeneity of this disease.^5^^,^^10^ Therefore, it is crucial to establish the underlying molecular mechanisms underlying lung cancer and to develop new, effective diagnostic biomarkers and treatment strategies to improve patient survival.^11^

Long noncoding RNAs (lncRNAs) are a class of RNA molecules with a length of more than 200 nucleotides and play a vital role in many aspects of cancer biology.^12^, ^13^, ^14^, ^15^ Studies have shown that lncRNAs are often highly dysregulated in tumors,^16^, ^17^, ^18^, ^19^ affecting cell proliferation, cell cycle progression, and apoptosis, and thus integrally involved in the development of cancer.^20^ lncRNAs can regulate gene expression through several different mechanisms, including chromatin modification, transcription, and post-transcriptional processing.^14^^,^^21^ We recently generated transcriptome data using next-generation RNA sequencing (RNA-seq) to reveal noncoding RNA expression patterns in lung cancer.^22^ Mining this high-density RNA-seq data, we identified several dysregulated lncRNAs in lung cancer, including *LINC00857*,^22^ *MIR22HG*,^16^ *LINC00152*,^23^ and *FAM83H-AS1*,^24^ which may have potential as new diagnostic or prognostic markers, as well as therapeutic targets for lung cancer.

*LINC00857* was one of the top dysregulated lncRNAs in lung cancer,^22^ both highly expressed and its upregulation related to poor patient survival in lung cancer. *LINC00857* was found to regulate cell proliferation, migration, invasion, and tumor growth in lung cancer^22^ and recently also shown to play an oncogenic role in gastric, bladder, liver, and esophageal cancers.^25^, ^26^, ^27^, ^28^, ^29^ The molecular mechanisms of its role in cancer biology, however, remain poorly understood. In this study, we demonstrate that *LINC00857* regulates cell proliferation in lung cancer cells with various genomic alterations and also found that *LINC00857* can affect cell death signaling, including both cell apoptosis and autophagy. Mechanistically, we discovered that *LINC00857* can interact with the Y-box binding protein 1 (YBX1) protein and protect it from undergoing proteasomal degradation. We thus show that *LINC00857* regulates apoptosis and autophagy through YBX1-MET and phosphor-AMP-activated protein kinase (p-AMPK)a signaling.

## Results

### *LINC00857* Knockdown Impairs Cell Proliferation in Lung Cancer Cells with Different Genomic Alterations

**Figure 1:**
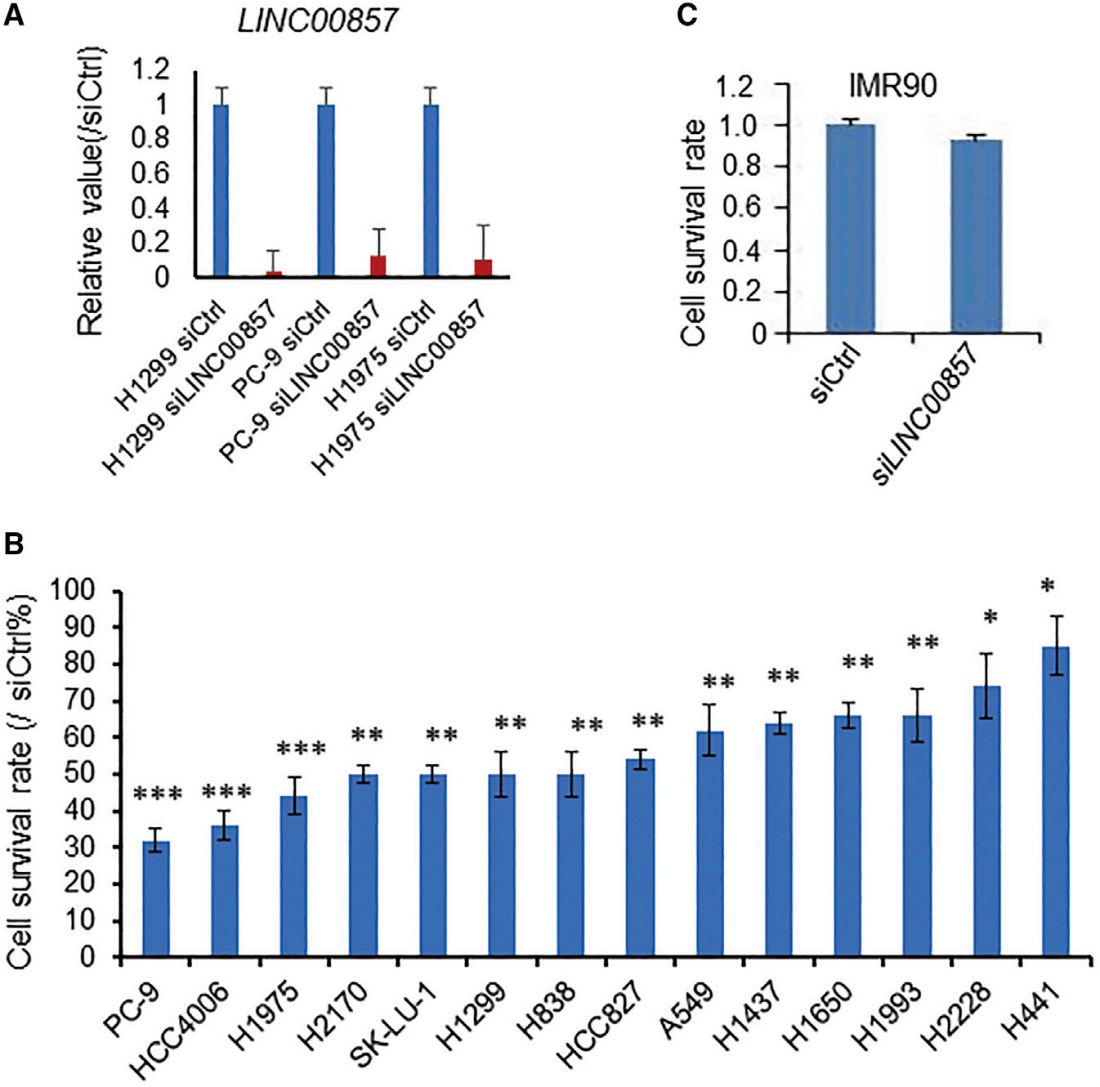
*LINC00857* Knockdown Impairs Cell Proliferation (A) *LINC00857* siRNA knockdown efficiency on 3 lung cancer cell lines mainly used in this study. (B and C) Effect of cell proliferation (cell survival rate) in lung tumor cells and lung fibroblast cell IMR-90 after *LINC00857* knockdown by siRNA (10 nM) measured by WST-1 (at 120 h). Cell survival rate is normalized to nontargeting control siRNA (siCtrl). Values represent the mean ± SD from three independent experiments. ∗p < 0.05, ∗∗p < 0.01, ∗∗∗p < 0.001.

In our recent publication, knocking down *LINC00857* reduced tumor cell proliferation, colony formation, migration, invasion *in vitro*, and tumor growth *in vivo*.^22^ In that study, we only used H1299 and H838 cell lines, thus to investigate whether *LINC00857* has a broad effect on cancer cell growth; here, we tested 14 lung cancer cell lines representing different histological subtypes (including 13 ADs and 1 SCC) and different genomic alterations, including the *EGFR*, *KRAS*, *TP53*, *MET*, and *LKB11* mutation status (Table S1) using the WST-1 cell proliferation assay following *LINC00857* knockdown. The efficiency of small interfering RNA (siRNA)-mediated knocking down of *LINC00857* was 80%–95% (Figure 1A). We found that the cell survival rate was from 32% to 85% on these 14 tested NSCLC cell lines upon *LINC00857* knockdown with siRNAs, indicating that *LINC00857* could affect cell proliferation regardless of the *KRAS*, *EGFR*, *MET*, *LKB11*, and *TP53* mutation status and histological subtypes (Figure 1B). Cell proliferation was not affected by *LINC00857* knockdown in the normal lung fibroblast cell IMR-90 (Figure 1C), suggesting that the role of *LINC00857* may be less relevant in normal cells.

### *LINC00857* Knockdown Induces Apoptosis and Autophagy

**Figure 2:**
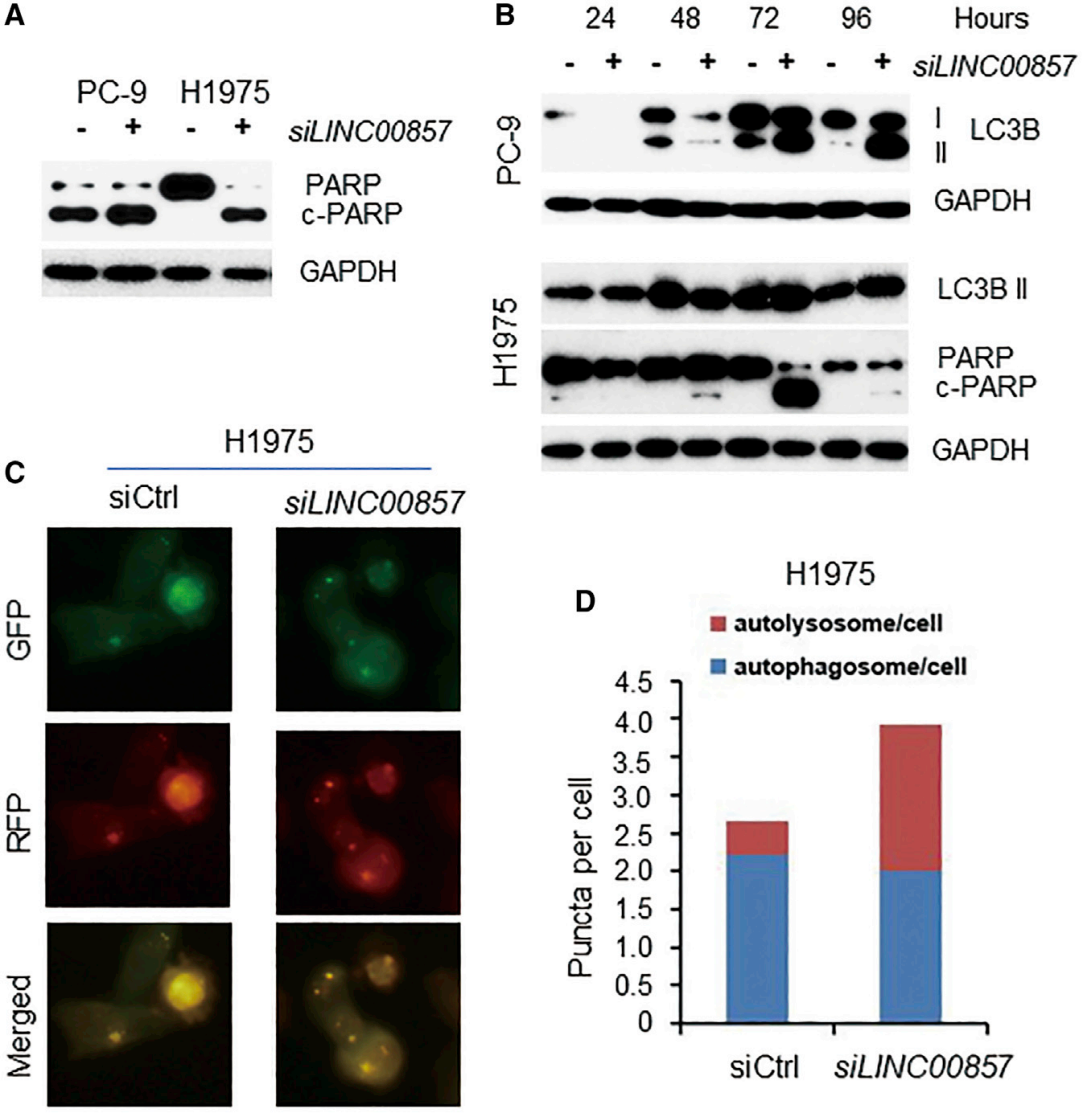
*LINC00857* Knockdown Induces Apoptosis and Autophagy (A) Cleaved PARP was increased after *LINC00857* knockdown by siRNA measured by western blot (at 72 h). (B) Autophagy marker (LC3B I to II conversion) was induced at 72 h for both PC-9 and H1975 after *LINC00857* knockdown by siRNA. Nontargeting siRNA (NT) as control (indicated by −). (C) H1975 cells were treated with siRNAs for 48 h and infected with Premo Autophagy Tandem Sensor RFP-GFP-LC3B for 24 h. Live cells were visualized with a fluorescence microscope. (D) Autophagosomes and autolysosomes in each 200× field (from image C) were counted; at least 100 cells were counted for each siRNA treatment cell line.

Since we found that *LINC00857* could affect cell proliferation through cell cycle regulation,^22^ we investigated whether *LINC00857* might affect cell death signaling. We measured the apoptosis marker cleavage PARP (c-PARP) protein and the autophagic marker LC3B, as well as the autophagic flux assay using the Premo Autophagy Tandem Sensor red fluorescent protein (RFP)-GFP-LC3B. We found that the apoptosis marker c-PARP was significantly increased at 72 h on H1975 and PC-9 cells after *LINC00857* knockdown (Figures 2A and 2B). We also found that c-PARP was induced on H1299 and H2228 cells (Figure S1). The autophagy marker LC3B I to II conversion (II/I ratio) was increased at 72 h (Figure 2B), and the puncta of autolysosome was also increased (Figures 2C and 2D) after *LINC00857* knockdown. These results indicated that *LINC00857* is not only involved in cell proliferation but also in both apoptosis and autophagy, although how *LINC00857* regulates cell death is unknown.

### *LINC00857* Interacts with the YBX1 Protein and Protects It from Proteasomal Degradation

**Figure 3:**
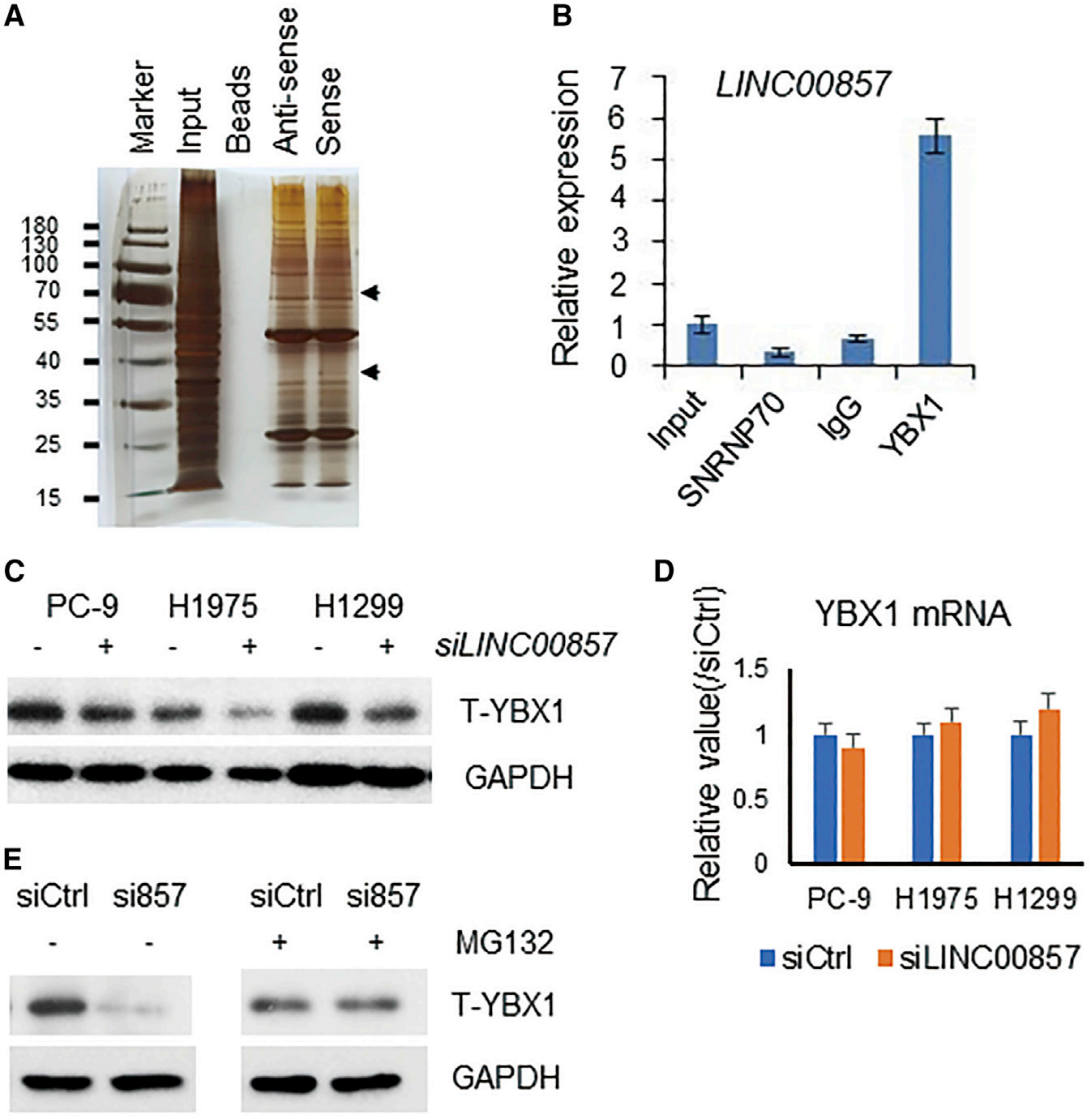
*LINC00857* Interacts with the YBX1 Protein (A) Silver staining of SDS-PAGE showing *LINC00857*-interacting proteins from *LINC00857* RNA pull-down assay. The different protein bands between the antisense group and sense group were indicated with arrows. They were submitted for mass spectrometry (MS) analysis. An extra band between 35 and 40 kD in *LINC00857* binding proteins was identified as YBX1 by MS. (B) Real-time PCR confirmed that *LINC00857* was accumulated in the YBX1-precipitated protein sample using the RIP assay in H1299 cell. (C) The YBX1 protein level was decreased after *LINC00857* knockdown with siRNA. (D) Real time PCR showing YBX1 mRNAs was not changed after *LINC00857* knockdown by siRNA as compared to control at 48 h. (E) *LINC00857* prevented YBX1 protein from proteasome degradation in PC-9 cells.

To explore the molecular mechanisms underlying the oncogenic activity of *LINC00857*, we sought to use RNA pull-down assays and mass spectrometry (MS) to identify proteins interacting with *LINC00857*. Silver staining of SDS-PAGE shows *LINC00857*-interacting proteins from the *LINC00857* RNA pull-down assay. The different protein bands between the antisense group and sense group were submitted for MS analysis (Figure 3A). Among the MS-identified proteins, YBX1 and CA6 (carbonic anhydrase 6) had the highest frequency and identity score, which indicated that *LINC00857* might bind to these proteins. To further confirm the interaction between *LINC00857* and YBX1, we performed a RNA immunoprecipitation (RIP) assay, in which the RNA-YBX1 complex was immunoprecipitated using a YBX1 antibody. The amount of *LINC00857* RNA in the coprecipitate was then measured by quantitative real-time PCR. Compared with the immunoglobulin G (IgG)-bound sample, the YBX1 antibody-bound complex had a significant increase in the amount of *LINC00857* RNA (Figure 3B), indicating that *LINC00857* may directly bind to the YBX1 protein. Further, we found that the YBX1 protein level was decreased after *LINC00857* knockdown with siRNA (Figure 3C), but the YBX1 mRNA level was not changed (Figure 3D), suggesting that *LINC00857* regulated YBX1 at the protein level. We found that the YBX1 protein was decreased more in *LINC00857* siRNA treatment cells as compared to control after treatment with protein synthesis inhibits reagent cycloheximide (CHX) (Figure S2). As MG132 is a proteasome inhibitor, we asked if *LINC00857* affected YBX1 protein degradation via the proteasome. As expected, we found that in the presence of MG132, YBX1 protein expression in the *LINC00857* knockdown cells was markedly increased and reached a level that was comparable to that in the control-treated cells (Figure 3E). These results indicated that *LINC00857* interacted with the YBX1 protein and prevented it from proteasomal-mediated degradation. YBX1 appears to be an important factor in lung cancer, and we previously reported that lung cancer cell proliferation was decreased after siRNA knockdown of YBX1, indicating that YBX1 may play an oncogenic role in lung cancer.^16^

### *LINC00857* Knockdown Blocks YBX1 Translocation from the Cytoplasm to the Nucleus

**Figure 4:**
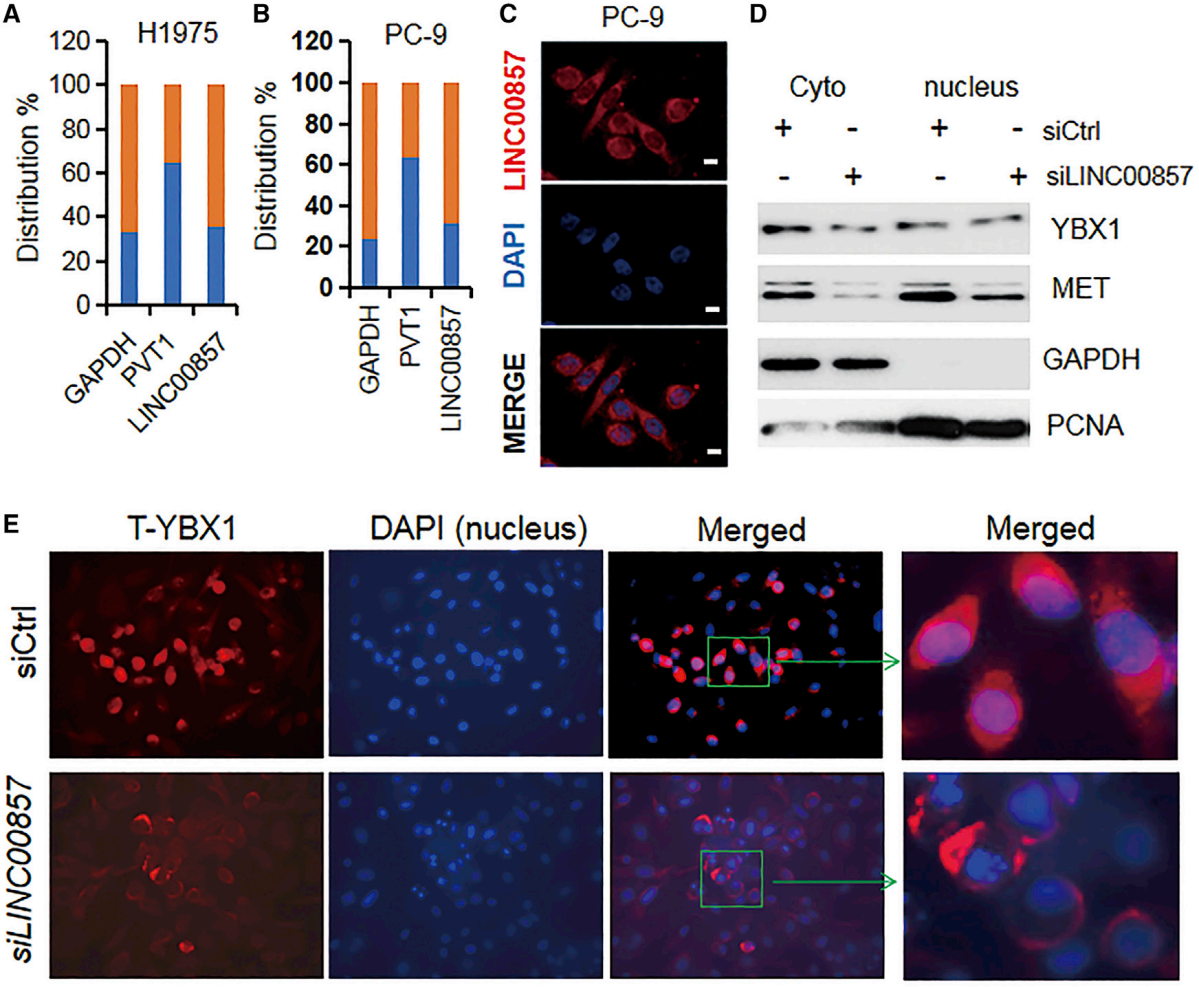
*LINC00857* Cell Location and Knockdown Block YBX1 Nuclear Translocation (A and B) Quantitative real-time PCR showing the nuclear and cytoplasmic fractions of *LINC00857* in H1975 and PC-9 cells. *LINC00857* is mainly in the cytoplasm (>60%–70%). GAPDH is used as a cytoplasmic control and PVT1) lncRNA as a nuclear control. Red, cytoplasmic; blue, nuclear. (C) RNA immunofluorescence staining (RNA fluorescence *in situ* hybridization [FISH]) showing *LINC00857* (red) located mainly in the cytoplasm in PC-9 cells. Bars, 40 μm. (D) Western blot indicates that YBX1 and MET proteins are present in both cytoplasmic and nuclear fractions. (E) YBX1 immunofluorescence staining in PC-9 cells. Red indicates YBX1 protein expression. Right image is magnified from left merged images.

In order to determine the cellular localization of *LINC00857*, we performed quantitative real-time PCR from RNA isolated from total, nuclear, and cytoplasmic fractions and found that *LINC00857* was primarily located in the cytoplasmic (60%–70%) and nuclear fractions (30%–40%) (Figures 4A and 4B). Immunofluorescence staining of *LINC00857* further confirmed that *LINC00857* is mainly located in the cytoplasm (Figure 4C). The YBX protein family has three members: YBX1, YBX2, and YBX3. The CSD (cold shock domain) of these three family members is more than 90% identical, yet there is no significant homology in the other parts of the molecules.^30^ YBX1 was reported to be expressed in both the cytoplasm and nucleus.^30^ We found that YBX1 protein is present in both cytoplasm and nuclear fractions by western blot, and YBX1 protein expression was decreased mainly in cytoplasm upon *LINC00857* knockdown (Figure 4D). The expression status and function of YBX2 and YBX3 were not clear. YBX1 was found to accumulate in the centrosome during the mitotic phase.^31^ YBX1 transitions from the cytoplasm to the nucleus in the following cases: at G1/S-phase interface, treatment with UV radiation, DNA-damaging agents, upon oxidative stress hyperthermia, and interaction with SRp30c and p53.^30^ We performed YBX1 immunofluorescence staining on PC-9 cells after *LINC00857* knockdown and found that YBX1 protein was present in both the cytoplasm and nucleus but accumulated in the nucleus at mitosis in control cells (rounded-up cells) (Figure 4E) and was consistent with previous reports.^31^^,^^32^ Importantly, we found that the nuclear total (T)-YBX1 protein was significantly decreased upon *LINC00857* knockdown, suggesting that *LINC00857* may not only affect T-YBX1 degradation but also affect YBX1 nuclear translocation.

### *LINC00857* or YBX1 Knockdown Decreases Oncogenic MET Expression

**Figure 5:**
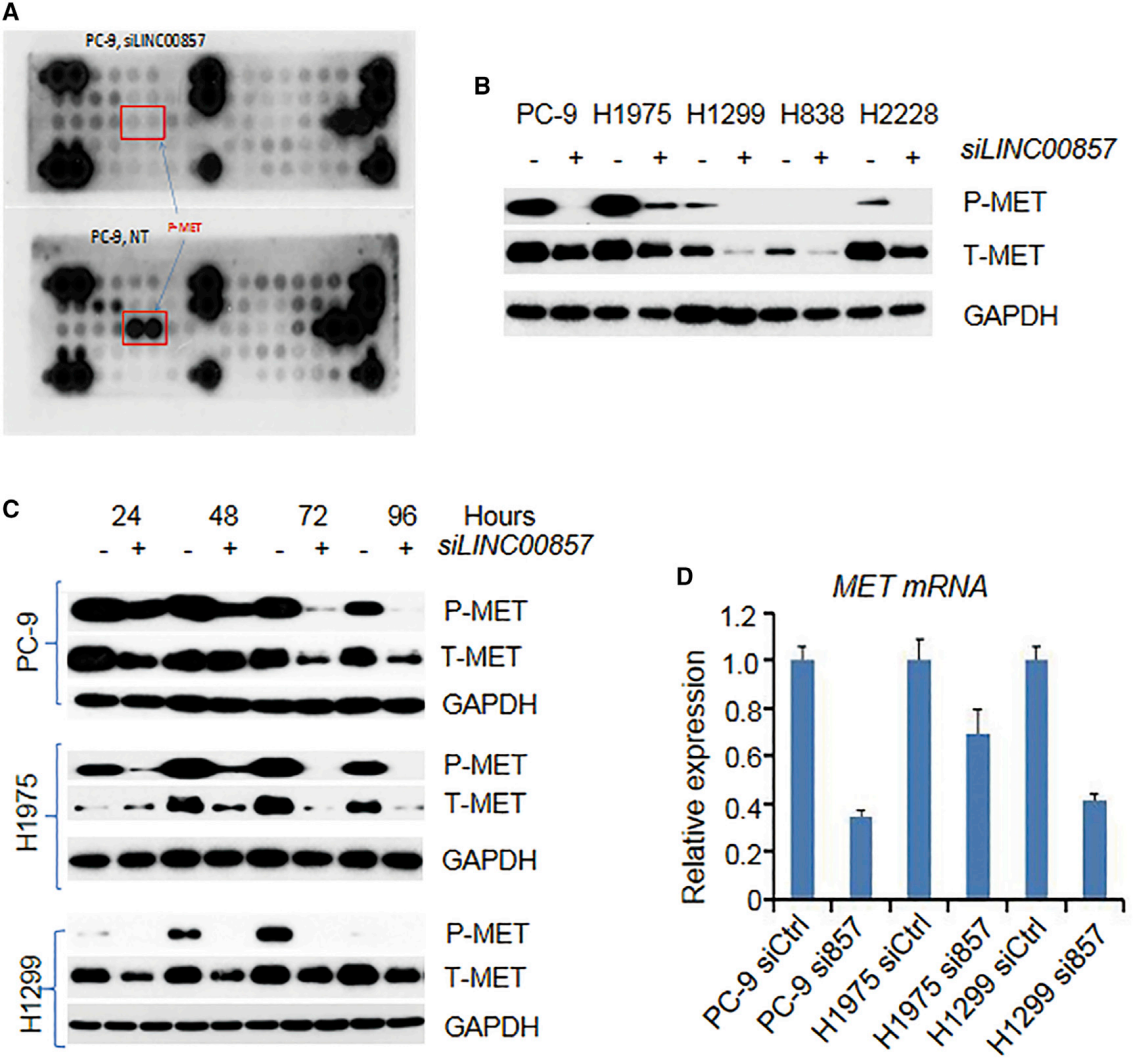
*LINC00857* Knockdown Decreases Oncogenic MET Expression (A) Receptor tyrosine kinase phosphorylation antibody array indicated that MET is the most significantly decreased protein after *LINC00857* knockdown by siRNA (10 nM at 72 h) as compared to control siCtrl in the PC-9 cell. (B) MET protein was decreased after *LINC00857* knockdown with siRNA in 5 cell lines by western blot. (C) MET protein was decreased after *LINC00857* knockdown with siRNA at 24- to 96-h time points by western blot. (D) MET mRNA was decreased after *LINC00857* siRNA treatment.

To uncover the potential proteins or pathways affected by *LINC00857*, we performed p-kinase protein antibody array and receptor tyrosine kinase phosphorylation antibody array analyses, which include more than 100 proteins covering most of the cancer-related pathways. Among these proteins, we found that the p-MET was the most significantly decreased protein after *LINC00857* knockdown in PC-9 lung cancer cells (Figure 5A). We then performed western blot analyses that confirmed that both p-MET and T-MET were decreased upon *LINC00857* knockdown in five lung cancer cell lines (Figure 5B), and the decrease in MET occurred as early as 24 h after *LINC00857* siRNA treatment (Figure 5C). With the use of quantitative real-time PCR, we found that MET mRNA was also decreased upon LINC00857 knockdown (Figure 5D). This suggests that *LINC00857* regulation of MET expression may be at the transcription level.

As a master regulator of cancer cell biology, YBX1 is involved in all of Hanahan’s “hallmarks of cancer.”^33^^,^^34^ The YBX1 protein performs its functions both in the cytoplasm (as an RNA binding protein) and in the cell nucleus (as a transcription factor).^30^^,^^35^ Nuclear YBX1 has been reported to promote MET expression by directly binding to the MET promoter in basal-like breast cancers.^36^ Consistent with this, we have previously confirmed that both MET protein and mRNA were decreased after YBX1 knockdown in lung cancer cells.^16^ From these results, we anticipated that *LINC00857* interacts with YBX1 and transports YBX1 to the nucleus, where as a transcription factor, YBX1 regulates MET expression.

### MET Knockdown Decreases Cell Proliferation and Induces Apoptosis and Autophagy

**Figure 6:**
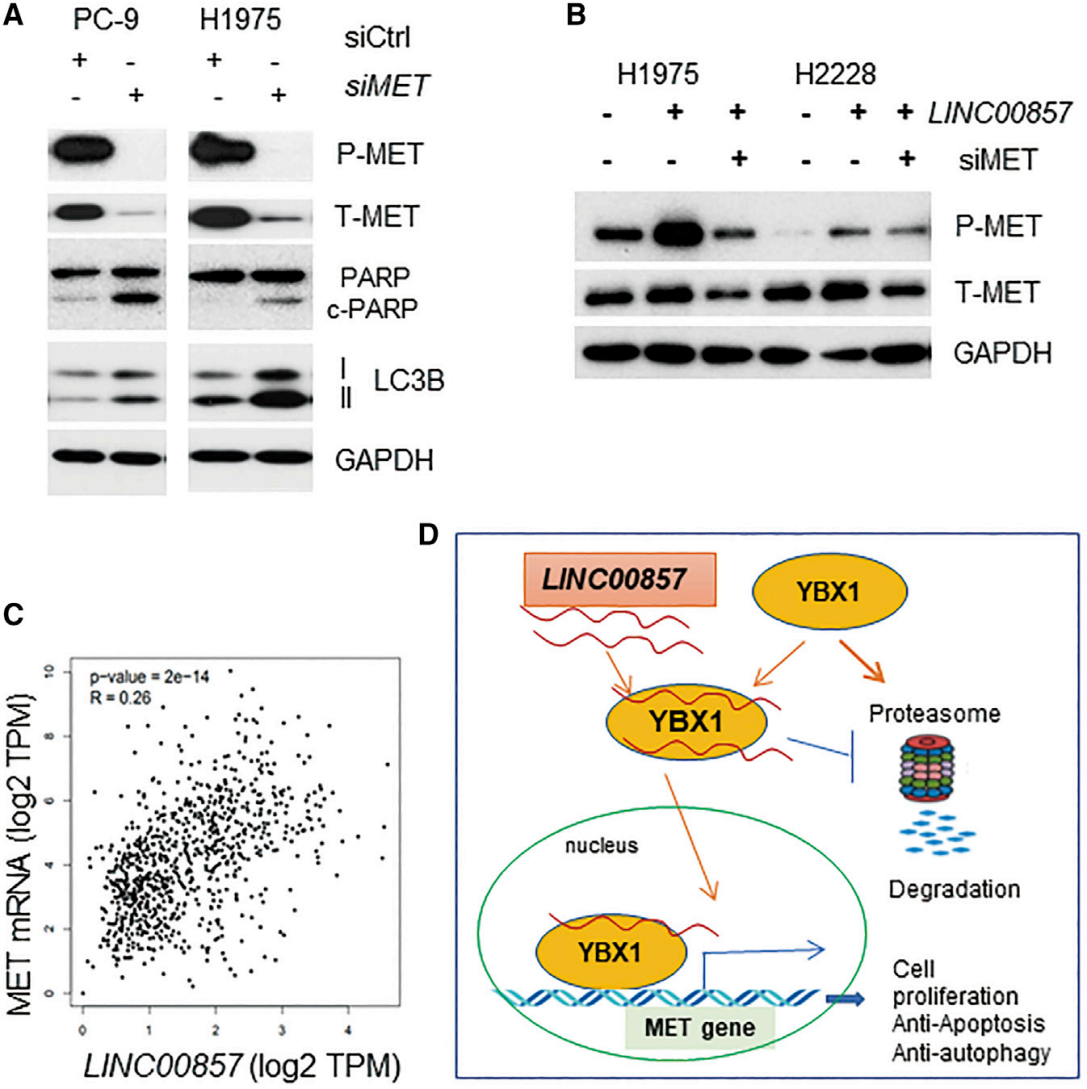
MET Knockdown Induces Apoptosis and Autophagy (A) Apoptosis (measured by cleaved PARP) and autophagy (measured by LC3B) were induced after MET knockdown. (B) MET protein was induced by overexpression of *LINC00857*-pcDNA-DEST53 transfection. The pcDNA-DEST53 vector was used as a control. (C) MET was significantly, positively correlated with *LINC00857* in LUAD. (D) Schematic of *LINC00857* in stabilizing YBX1 and promoting MET expression. In cells with higher *LINC00857* levels, *LINC00857* binds and prevents YBX1 from proteasome degradation and promotes MET expression by binding to the MET promoter. Upon *LINC00857* knockdown, YBX1 is degraded and blocks YBX1 nucleus translocation.

We have previously found that cell proliferation was decreased in lung cancer cells after MET knockdown with siRNA.^16^ Here, we found that apoptosis and autophagy were also induced by MET knockdown (Figure 6A), suggesting that MET may play an important role in *LINC00857* regulation of cell growth, apoptosis, and autophagy. Further, we found that the MET protein could be increased by *LINC00857* overexpression (Figure 6B), which further confirms that MET is regulated by *LINC00857*. With the use of the GEPIA (Gene Expression Profiling Interactive Analysis) dataset (http://gepia.cancer-pku.cn/), we found that MET mRNA was significantly, positively correlated with *LINC00857* in lung AD (LUAD) (Figure 6C). Taken together, it appears that *LINC00857* interacts with the YBX1 protein and protects it from proteasomal degradation in the cytoplasm. Then *LINC00857* brings YBX1 from the cytoplasm to the nucleus where YBX1 binds to the promoter of MET and regulates MET expression. Thus, that *LINC00857* plays an oncogenic role may be via this YBX1-MET axis (Figure 6D).

### *LINC00857* Knockdown Increases the Expression of the p-AMPKa Protein

**Figure 7:**
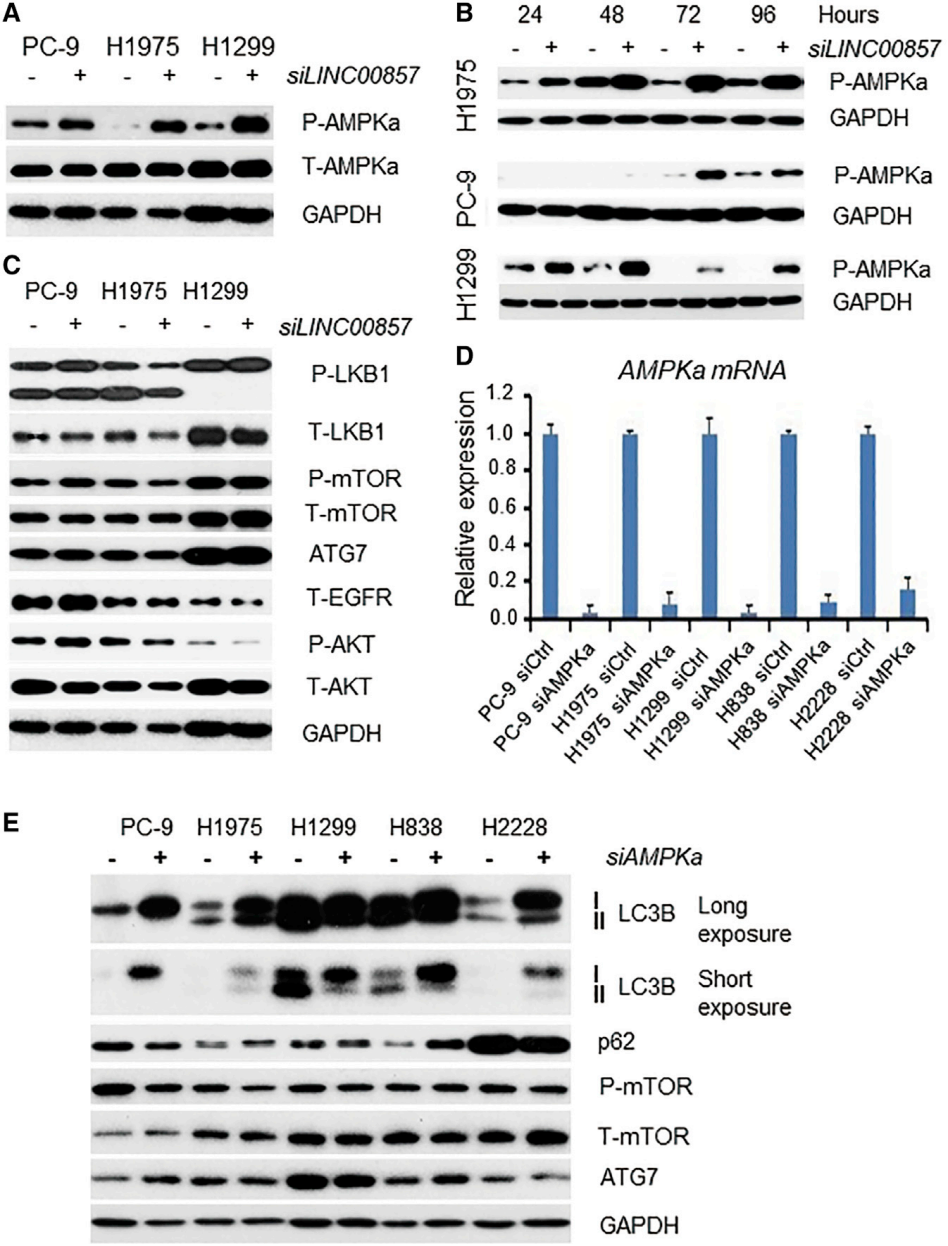
*LINC00857* Knockdown Increases the Expression of the Phosphor (p)-AMPKa Protein (A and B) p-AMPKa protein, not the total protein and mRNA, was significantly increased after *LINC00857* knockdown with siRNA in lung cancer cells and occurred as early as 24 h in H1975 and H1299 cells. (C) Other autophagy-related proteins, such as LKB1, mTOR, ATG7, as well as EGFR and AKT, were not changed significantly after *LINC00857* knockdown at 72 h. (D) AMPKa (RPKAA1 gene) mRNA knockdown efficiency using RPKAA1 siRNA at 48 h. (E) Autophagy was blocked (indicated by LC3B) after AMPKa knockdown. Changes of p62, mTOR, and ATG7 were not consistent among these cells.

In order to explore the mechanisms involved in *LINC00857* regulation of autophagy, we screened the expression of multiple autophagy-related proteins by western blot. We found p-AMPKa protein, not the total protein or mRNA, was significantly increased after *LINC00857* knockdown with siRNA in lung cancer cells, and this increased expression occurred as early as 24 h (Figures 7A and 7B). Other autophagy-related proteins, such as LKB1, mTOR, ATG7, EGFR, and AKT, were not significantly changed after *LINC00857* knockdown at 72 h (Figure 7C). The levels of p-AMPKa were not changed after YBX1 knockdown (data not shown). These results suggest that *LINC00857* regulation of p-AMPKa is independent of YBX1, LKB1, mTOR, and ATG7 molecules.

AMPK has critical roles in regulating both growth and reprogramming metabolism and has recently been connected to cellular processes, such as autophagy and cell polarity.^37^ AMPK controls autophagy at different points.^38^ In order to confirm whether AMPKa is required for autophagy regulation in lung cancer, we knocked down AMPKa (RPKAA1 gene) with siRNAs (Figures 7D and 7E), followed by western blot, using the autophagy marker LC3B. As shown in Figure 7E, the ratio of LC3B II/I (conversion from LC3B I to LC3B II) was significantly decreased, indicating that autophagy was blocked in all 5 lung cancer cell lines tested after AMPKa knockdown. Again, AMPKa downstream proteins, mTOR and ATG7, were not changed, indicating that these two proteins may be not required for AMPKa in regulating autophagy in these lung cancer cells.

### Crosstalk of *LINC00857*, AMPKa, and MET

**Figure 8:**
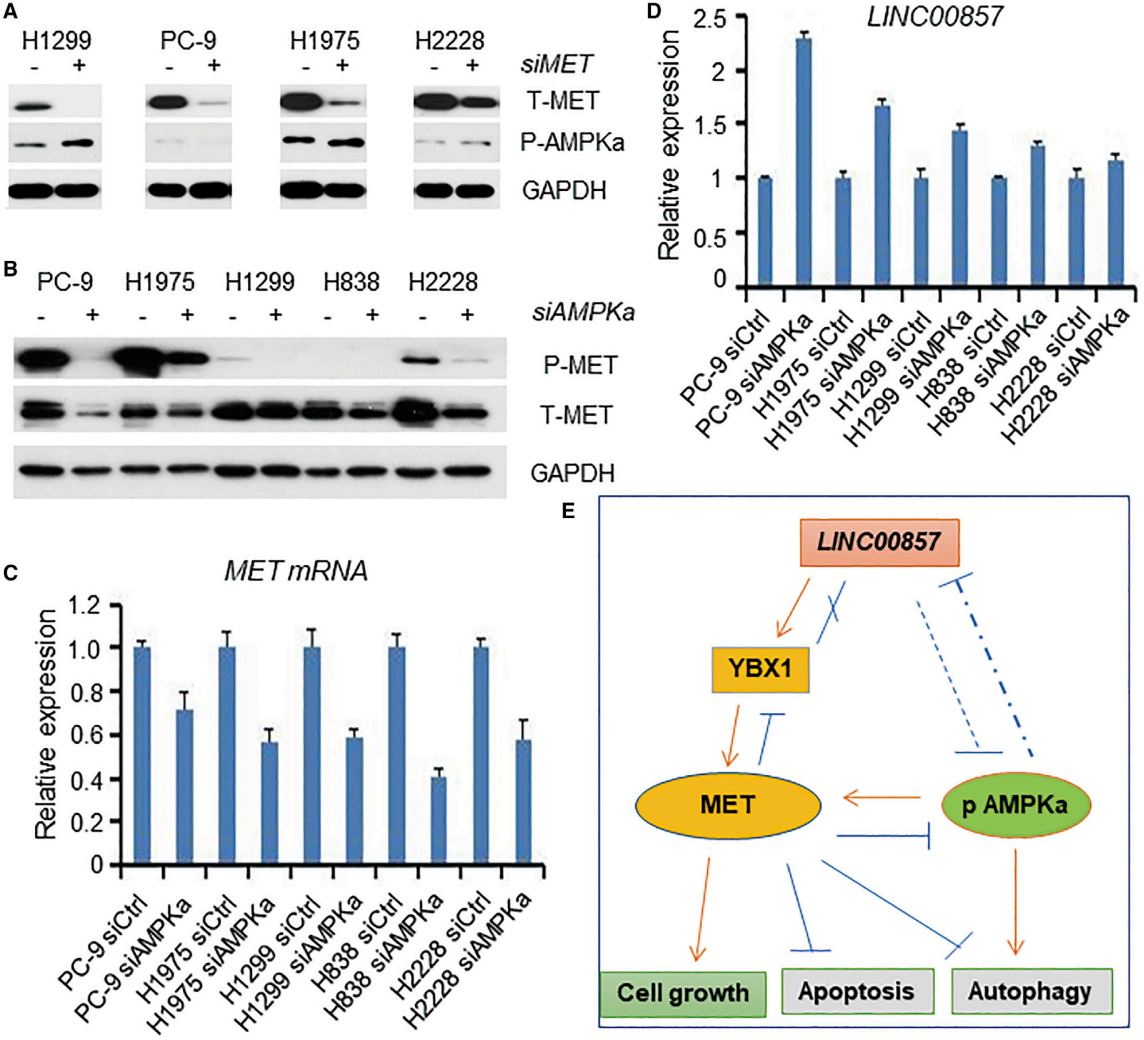
Crosstalk of *LINC00857*, AMPKa, and MET (A) p-AMPKa was slightly increased after MET knockdown with siRNAs at 72 h. (B and C) Both protein and mRNA of MET were significantly decreased after AMPKa (RPKAA1 gene) knockdown with siRNA at 72 h. (D) *LINC00857* was increased after AMPKa (RPKAA1 gene) knockdown with siRNA at 48 h. (E) Schematic of role of *LINC00857* in regulation of cell proliferation, apoptosis, and autophagy through YBX1-MET and AMPKa signaling.

From our preliminary studies, we have found that *LINC00857* regulates apoptosis and autophagy potentially via the YBX-MET axis and autophagy via p-AMPKa. In order to understand if there is crosstalk or feedback among these three major molecules, we performed knockdown of MET or AMPKa, followed by real-time PCR or western blot. We found that p-AMPKa protein was slightly increased in H1299 cells and in H1975 cells after MET knockdown (Figure 8A), yet no changes of AMPKa mRNA and *LINC00857* were observed (data not shown). Surprisingly, we found that both MET protein and mRNA were significantly decreased upon AMPKa knockdown (Figures 8B and 8C), indicating that a role of AMPKa in regulating MET expression is at the transcriptional level, although currently, the mechanism is unknown. *LINC00857* expression was increased after AMPKa knockdown (Figure 8D), indicating that there may be a feedback between AMPKa and *LINC00857*. Taken together, *LINC00857* regulation of apoptosis and autophagy may be through two separate signaling mechanisms (Figure 8E).

## Discussion

Lung cancer is a complex disease associated with a variety of genetic mutations, epigenetic alterations, chromosomal translocations, deletions, and amplifications.^39^^,^^40^ The poor outcomes of lung cancer patients may be partially due to the complicated molecular mechanisms underlying the cancer progression, as well as lack of early diagnostic biomarkers and therapeutic targets.^41^, ^42^, ^43^ The aberrant expression of lncRNAs is a molecular phenotype of cancers^19^^,^^44^ and involved in each of the hallmarks of cancer phenotypes, including proliferation, growth suppression, motility, immortality, angiogenesis, and viability.^45^ The molecular mechanisms underlying these cancer phenotypes regulated by lncRNAs influence interactions with cellular macromolecules, including chromatin, proteins, mRNAs, or microRNAs (miRNAs).^45^ We have previously reported that *LINC00857* was upregulated in lung cancer tissues, and its increased expression was correlated with poor survival in patients with lung cancer.^22^ Further, we found *LINC00857* regulates cell proliferation and tumor growth via cell cycle regulation.^22^ In this study, we uncovered new mechanisms of the *LINC00857* role in lung cancer. We found that *LINC00857* can affect apoptosis and autophagy. *LINC00857* can interact with YBX1 protein and protect it from proteasomal degradation. YBX1-MET and p-AMPKa were the major signaling for *LINC00857* in regulating apoptosis and autophagy.

Numerous studies have established that lncRNAs can modulate cell proliferation, migration, invasion, drug resistance, apoptosis, and autophagy in cancer cells.^46^, ^47^, ^48^ *LINC00857* has been reported to play oncologic roles in several cancers.^22^^,^^25^, ^26^, ^27^, ^28^ For example, *LINC00857* regulates cell proliferation in lung, bladder, gastric, esophageal, and liver cancer.^22^^,^^25^, ^26^, ^27^, ^28^, ^29^ Knockdown of *LINC00857* sensitizes bladder cancer cells to cisplatin.^25^ *LINC00857* silencing represses hepatocellular carcinoma (HCC) cell epithelial-mesenchymal transition (EMT) phenotype.^27^ Recent reports indicate that *LINC00857* knockdown can induce apoptosis in HCC, esophageal AD, and LUAD;^27^, ^28^, ^29^ however, the underlying mechanisms remains unclear. In this study, we discovered that *LINC00857* knockdown could induce apoptosis, which may be via the YBX1-MET and AMPKa signaling pathways.

YBX1 functions as an RNA-binding protein and has been implicated in numerous cellular processes, including the regulation of transcription and translation.^30^^,^^49^ YBX1 regulates gene transcription and translation; on the other hand, YBX-1 is controlled by oncogenes or tumor-suppressor genes. Cell fate factor DACH1 can represses YBX1-mediated oncogenic transcription and translation.^50^ Several reports demonstrate that some lncRNAs plays oncogenic roles through interaction with YBX1.^16^^,^^51^, ^52^, ^53^, ^54^, ^55^ In this study, we found that *LINC00857* can bind and stabilize the YBX1 protein. *LINC00857* knockdown decreases YBX1 protein levels (not mRNA level) and can affect YBX1 protein nuclear translocation. Experiments of YBX1-associated ubiquitination in control and *LINC00857* knockdown cells treated with MG132 may be needed in the future. Notably, loss of *LINC00857* or YBX1 decreases MET expression at both the mRNA and protein levels. Previously, we^16^ and others^36^ have reported that YBX1 could regulate MET expression via binding to the MET promoter region. These results suggest that *LINC00857* may participate in tumorigenesis functions, including apoptosis and autophagy, through the *LINC00857*-YBX1-MET axis.

Autophagy is a highly evolutionarily conserved, lysosomal-dependent degradation pathway that is widely present in eukaryotic cells.^56^ Abnormal autophagy function is associated with many diseases, such as cancer, neurodegenerative diseases, muscle diseases, diabetes, pathogenic microbial infections, etc.^57^ Autophagy plays a dual role in the development of lung cancer. On one hand, autophagy can eliminate harmful substances in the body and inhibit the formation of lung tumors, yet it can also alter environmental homeostasis and affect the survival and metastasis of tumor cells.^58^ Tumor cells can also acquire energy through autophagy to maintain their rapid proliferation. Several studies have demonstrated that lncRNAs could regulate autophagy underlying different mechanisms.^15^^,^^47^^,^^59^ In this study, we found that autophagic marker LC3B II and an autolysosome were induced after *LINC00857*, MET, or p-AMPKa knockdown, which supports that *LINC00857* affects autophagy via YBX1-MET and p-AMPKa signaling.

AMPK can be activated by various types of metabolic stress that lead to ATP depletion, such as conditions of low nutrient supply, prolonged exercise, or via an increase in intracellular Ca^2+^ concentration. The upstream kinases, LKB1 and calcium/calmodulin-dependent protein kinase β (CAMKKβ), activate AMPK by phosphorylating Thr172 in the activation loop of the catalytic α-subunit.^60^ AMPK has critical roles in regulating growth and reprogramming metabolism and has recently been connected to cellular processes, such as autophagy and cell polarity.^37^ AMPK controls autophagy at different steps.^38^ Several lncRNAs were reported to be involved in AMPK regulation.^61^^,^^62^ Depletion of lncRNA NBR2 attenuates energy stress-induced AMPK activation, resulting in unchecked cell cycling, an altered apoptosis/autophagy response, and increased tumor development *in vivo*.^63^ In order to confirm if AMPKa is required for autophagy regulation in lung cancer, we knocked down AMPKa (RPKAA1 gene) with siRNAs, followed by western blot, using the autophagy marker LC3B. As shown in Figure 7E, the ratio of LC3B II/I (conversion from LC3B I to LC3B II) was significantly decreased, indicating that autophagy was blocked in all five lung cancer cell lines tested after AMPKa knockdown. Again, AMPKa downstream proteins, mTOR and ATG7, were not changed, indicating these two proteins may be not required for AMPKa regulation of autophagy in these lung cancer cells. We conclude that p-AMPKa is required for *LINC00857* to regulate autophagy in lung cancer cells. Because the mechanism of how *LINC00857* regulates p-AMPK is still not clear, further characterization is needed.

In summary, *LINC00857* can interact with YBX1 protein and prevent it from proteasome degradation. *LINC00857* can help YBX1 nuclear translocation and regulate MET expression. lncRNA *LINC00857* silencing can impair cell proliferation and induce apoptosis and autophagy in lung cancer, which may be through YBX1-MET and p-AMPKa signaling. Our data reveal that *LINC00857* plays a critical role in cell survival and death signaling and may provide a strategy for using *LINC00857* as a potential biomarker and a therapeutic target for lung cancer.

## Materials and Methods

### Cell Culture

Human cell lines PC-9, H1299, H1975, H838, and H2228 were obtained from the American Type Culture Collection. All cells were cultured in RPMI-1640 medium (Gibco, Carlsbad, CA, USA). All media were supplemented with 10% fetal bovine serum (Gibco-BRL, Gaithersburg, MD, USA) and maintained in a 37°C incubator with a humidified atmosphere containing 5% CO_2_.

### Cell Proliferation Assays

Cells were plated at a density of 1,000 cells per well in a 96-well plate. *LINC00857* siRNA and control siRNA were added at 24 and 48 h. Cell proliferation was measured using a WST-1 reagent (Roche, Mannheim, Germany) from 96 to 120 h after siRNA transfection, according to the manufacturer’s instructions. All experiments were repeated three times. siRNA sequences used in this study were summarized in Table S2.

### RNA Isolation and Quantitative Real-Time PCR

Total RNA from human tissues and cultured cells was isolated using TRIzol reagent (Invitrogen, Carlsbad, CA, USA) following the manufacturer’s protocol. Then 2 μg of RNA was reverse transcribed into complementary DNA (cDNA) using Moloney murine leukemia virus (M-MLV) Reverse Transcriptase (Invitrogen). Quantitative real-time PCR was performed on the Applied Biosystems 7500 Real-Time PCR System (Applied Biosystems, Foster City, CA, USA) using SYBR Premix Ex Taq II (Takara, Dalian, China). The expression of lncRNA and mRNAs was normalized to glyceraldehyde 3-phosphate dehydrogenase (*GAPDH*). The gene-specific primer sequences used were summarized in Table S3.

### Western Blotting

Cells were harvested 72 h after siRNA transfection. Lysis, electrophoresis, and target protein visualization were performed, as described previously.^22^ Total cell lysates were prepared with sample buffer and boiled at 95°C for 5 min. The samples were transferred to SDS-PAGE at 80 V for 3 h and then transferred to polyvinylidene fluoride (PVDF) membranes for another 3 h. After incubation with specific antibodies for T-MET, p-MET, T-YBX1, PARP, LC3B, T-AMPKa, p-AMPKa, T-LKB1, p-LKB1, T-mTOR, p-mTOR, ATG7, T-EGFR, T-AKT, p-AKT, proliferating cell nuclear antigen (PCNA), and GAPDH at 4°C overnight, the membranes then were washed by 1% Tris-buffered saline-Tween 20 (TBST) three times, incubated with secondary antibodies for 1 h, developed using enhanced chemiluminescence (ECL), and exposed to X-ray film. More detailed information of antibodies used in this study was summarized in Table S4.

### Cytoplasmic and Nuclear RNA Isolation

Cytoplasmic and nuclear RNA were isolated and purified using the Cytoplasmic and Nuclear RNA Purification Kit (Norgen, Belmont, CA, USA), following the manufacturer’s protocol.

### RNA Pull-Down Assays

RNA pull-down assays were used to identify proteins interacting with *LINC00857*. Briefly, biotinylated full-length *LINC00857* or antisense transcript (negative control), synthesized by the TranscriptAid T7 High Yield Transcription Kit (Cat. K0441; Thermo Fisher Scientific, USA), was incubated with the protein lysate from H1299 cells, and the coprecipitated proteins were isolated with streptavidin-agarose beads by the RiboTrap Kit (Cat. RN1012; MBL International, Japan). The RNA-associating proteins were resolved on SDS-PAGE gel, and the specific bands of potential *LINC00857*-bound proteins were compared with an antisense transcript and determined by MS.

### RIP Assays

RIP experiments were performed using a Magna RIP RNA-Binding Protein Immunoprecipitation Kit (Cat. 17-701; Millipore, USA), according to the manufacturer’s instructions. The antibodies for RIP assays of YBX1 (Cat. ab12148, ab76149) were from Abcam (Cambridge, MA, USA).

### Autophagic Flux Measurement

Autophagic flux was measured by Premo Autophagy Tandem Sensor RFP-GFP-LC3 reagent (Life Technologies; P36239). Cells in 96-well plates were treated with *LINC00857* siRNA for 24 h, and RFP-GFP-LC3 reagent was added to each well at a concentration of 20 particles, according to the manufacturer’s instructions. Fluorescence images were captured after 24 h incubation using microscope, and the autophagosomes (yellow punctas in fusion images) and autolysosomes (red punctas in fusion images) were counted in at least 100 cells in a 200× field.

### Statistical Analysis

All statistical analyses were performed using Prism version 7 (GraphPad Software, CA, USA) and R software. All values are expressed as mean ± standard deviation (SD). Cell proliferation data were analyzed using Student’s t tests. All tests were 2 sided, and statistical significance was noted at p < 0.05. Three triplicate independent experiments were performed for cell biological assays, unless otherwise stated.

## Supplemental Information

Document S1. Tables S1–S4 and Figures S1 and S2

Document S2. Article plus Supplemental Information
