# Supplementary material for: LINC00857 Interacting with YBX1 to Regulate Apoptosis and Autophagy via MET and Phosphor-AMPKa Signaling
Source: Mol Ther Nucleic Acids. 2020 Oct 22;22:1164–75. doi: 10.1016/j.omtn.2020.10.025 (PMC7701017; doi:10.1016/j.omtn.2020.10.025)
Supplement: Document S1. Tables S1–S4 and Figures S1 and S2 [file mmc1.pdf]

OMTN, Volume *22*

Supplemental Information

LINC00857 Interacting with YBX1 to Regulate Apoptosis and Autophagy via MET and Phosphor-AMPKa Signaling

Wenmei Su, Lihui Wang, Huijie Zhao, Shengmin Hu, Yi Zhou, Chunfang Guo, Bin Wu, Lixia Li, Zhixiong Yang, David G. Beer, and Guoan Chen

***LINC00857* Interacting with YBX1 to Regulate Apoptosis and Autophagy via MET and Phosphor-AMPKa Signaling**

Wenmei Su[1*], Lihui Wang[2*], Huijie Zhao[3], Shengmin Hu[3], Yi zhou[3], Chunfang Guo[4], Bin Wu[1], Lixia Li[1], Zhixiong Yang[1], David G. Beer[4], Guoan Chen[3]

Supplemental Information

Table S1 Lung cancer cell lines with different genomic alterations

Cell Line	Subtype	*EGFR* mutation	*KRAS* mutation	*TP53* mutation	*MET* mutation	*LKB1* mutation	*LINC00857* expression	*LINC00857* siRNA on cell death
PC-9	AD	E746_A750del	WT	WT	WT	WT	5.2	68
HCC4006	AD	Del L747-E749	WT	WT	WT	WT	2.4	64
H1975	AD	L858R, T790M	WT	WT	WT	WT	3.8	56
H2170	SCC	WT	WT	R158G	WT	WT	4.5	50
SK-LU-1	AD	WT	G12D	H193R	WT	WT	2.8	50
H1299	AD	WT	WT	trancated	WT	WT	5.1	50
H838	AD	WT	WT	E62stop	p.I638L	loss	7.0	50
HCC827	AD	Del E746-A750	WT	WT	WT	WT	4.4	46
A549	AD	WT	G12S	WT	WT	Q37, loss	8.1	38
H1437	AD	WT	WT	R267P	WT	WT	5.3	36
H1650	AD	E746_A750del	WT	WT	WT	WT	7.2	34
H1993	AD	WT	WT	R209stop, C242W	amp	WT	3.0	34
H2228	AD	EML4-ALK fusion	WT	Q331stop	WT	WT	1.7	26
H441	AD	WT	G12V	R158L	p.S178S	WT	5.2	15

Note: Lung cancer cell lines used for cell proliferation tested with *EGFR*, *KRAS*, *TP53*, *MET*, or *LKB1* gene mutation status, *LINC00857* expression levels (FRPK value of RNA-seq, log2) and cell death rate.

Table S2 siRNA sequences used in this study:

Gene name	ID	sequence	Company
LINC00857	CTM-250373	Sense: 5' GAGAAAUGCUCAUGGGAAUUU	Dharmacon
MET	CTM-277344	Sense: 5' AGACAAGCAUCUUCAGUUAUU	Dharmacon
YBX1 #3	CTM-285106	Sense: 5' CGGCAAUGAAGAAGAUAAAUU	Dharmacon
AMPKa #1	CTM-313373	Sense: 5' GCAGAAGUAUGUAGAGCAAUU	Dharmacon
control siRNA #1	D-001810-01-20	ON-TARGETplus Nontargeting	Dharmacon

Table S3 Primer sequences used in this study:

gene name	Primer ID	Primer Sequence (5'->3')	product size
LINC00857	Lnc1 F	CCCCTGCTTCATTGTTTCCC	131
LINC00857	Lnc1 R	AGCTTGTCCTTCTTGGGTACT	
YBX1	YBX1 F	AAGGAGAAAAGGGTGCGGAG	113
YBX1	YBX1 R	CCTACGACGTGGATAGCGTC	
AMPKa	AMPKa F	CGGAGCCTTGATGTGGTAGG	183
AMPKa	AMPKa R	TTCATCCAGCCTTCCATTCTT	
MET	MET F	CAACCCGAATACTGCCCAGA	99
MET	MET R	CCGGGACACCAGTTCAGAAA	
GAPDH	GAPDH gcF	GTCAAGGCTGAGAACGGGAA	158
GAPDH	GAPDH gcR	AAATGAGCCCCAGCCTTCTC	

Table S4 Antibodies used in this study:

P-MET (Cell Signaling Technology, 3077)

T-MET (Cell Signaling Technology, 8198)

T-YBX1 (Santa Cruz Biothecnology, sc-398340)

T-YBX2 (Gene Tex, GTX116127)

T-YBX3 (Gene Tex, GTX77783)

P-AMPKa (Cell Signaling Technology, 2535)

T-AMPKa (Cell Signaling Technology, 5832)

P-LKB1 (Cell Signaling Technology, 3482)

T-LKB1 (Cell Signaling Technology, 3050)

P-mTOR (Cell Signaling Technology,2983T)

T-mTOR (Cell Signaling Technology,5536T)

ATG7 (Cell Signaling Technology, 8558)

T-EGFR (Cell Signaling Technology, 54359)

P-AKT (Cell Signaling Technology, 4056)

T-AKT (Cell Signaling Technology, 4691)

LC3B (Cell Signaling Technology, 2775)

SQSTM1/P62 (Cell Signaling Technology, 5114)

PCNA (Cell Signaling Technology, 2586)

GAPDH (Millipore Sigma, AB2302)

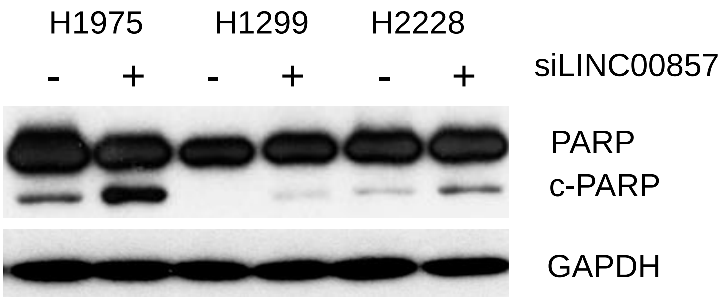

Figure S1 Western blot showing the cleaved PARP (c-PARP) were induced after LINC00857 knockdown by siRNA on 3 lung cancer cell lines.

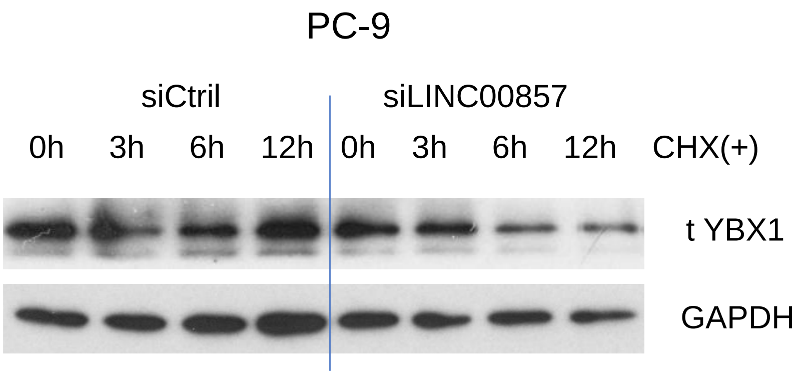

Figure S2 Western blot showing YBX1 protein was decreased more after LINC00857 knockdown by siRNA as compared to control at 6-12 hrs. Both were treated with protein synthesis inhibit regent cycloheximide (CHX) at 12.5 µg/ml.
